# Implication of thyroid function in periodontitis: a nationwide population-based study

**DOI:** 10.1038/s41598-021-01682-9

**Published:** 2021-11-11

**Authors:** Eyun Song, Min Jeong Park, Jung A. Kim, Eun Roh, Ji Hee Yu, Nam Hoon Kim, Hye Jin Yoo, Ji A. Seo, Sin Gon Kim, Nan Hee Kim, Sei Hyun Baik, Kyung Mook Choi

**Affiliations:** Division of Endocrinology and Metabolism, Department of Internal Medicine, Korea University College of Medicine and School of Medicine, Seoul, Korea

**Keywords:** Endocrinology, Thyroid diseases, Risk factors

## Abstract

Possible links between periodontitis and various cardiometabolic and autoimmune diseases have been advocated on the basis of chronic inflammation or oxidative stress. However, the association between periodontitis and thyroid dysfunction is under-researched. Participants without previous thyroid disease or ongoing thyroid-related medication were included from a nationwide population-level survey. Participants were categorized into tertiles of thyroid stimulating hormone (TSH) levels (first tertile < 1.76 mIU/L; second tertile 1.76–2.83 mIU/L; third tertile > 2.83 mIU/L), and periodontal condition was assessed using the Community Periodontal Index. Of the total of 5468 participants, 1423 had periodontitis (26%). A significant difference in the weighted prevalence of periodontitis according to TSH tertiles was observed, with the highest prevalence in the first tertile (26.5%) and the lowest prevalence in the third tertile (20.9%, *p* = 0.003). Subjects in the first TSH tertile had higher odds for periodontitis than those in the third tertile (OR 1.36, 95% CI 1.10–1.68; *p* for trend = 0.005) after adjusting for covariates. This association was consistent across subgroups and within sensitivity analyses among subjects without specific factors affecting thyroid function or diseases reported to be related to periodontitis. The present study demonstrated that low TSH levels were associated with significantly higher odds for periodontitis.

## Introduction

The inflammatory process underpins the etiology of a wide variety of systemic diseases, ranging from metabolic to psychological disorders^1^. Chronic inflammation can lead to hypertension, dyslipidemia, cardiovascular disease (CVD), type 2 diabetes mellitus (DM), non-alcoholic fatty liver disease (NAFLD), chronic kidney disease (CKD), and neurodegenerative conditions. These conditions are the primary causes of morbidity and mortality worldwide, accounting for more than 50% of all deaths^2,3^.

Periodontitis is one of the most common chronic infectious diseases in humans, occurring as a result of dysregulation of the host immune response triggered by subgingival microorganisms^4,5^. It is highly prevalent worldwide with severe periodontitis affecting 7.4% of the global population, posing a significant economic burden and major public health challenge^6,7^. The potential connections between periodontal disease and systemic disorders have attracted considerable interest in recent decades. In fact, a large body of evidence supports the association between periodontitis and various diseases, including CVDs^8–10^, DM^11,12^, metabolic syndrome^13^, obesity^14^, NAFLD^15^, CKD^16^, osteoporosis^17,18^, rheumatoid arthritis^19^, Alzheimer's disease^20^, and Parkinson’s disease^21^. Inflammation (mediated in part by an increase in levels of the serum C-Reactive Protein and inflammatory cytokines tumor necrosis factor-α [TNF-α], interleukin-1 [IL-1], and IL-6), oxidative stress, and endothelial dysfunction have been proposed as the underlying mechanisms for the interrelationship between periodontitis and these diseases^22–27^.

The thyroid is a typical organ within which organ-specific autoimmune disease and chronic inflammation occur frequently. Thyroid hormones play an important role in oxidative stress and inflammation in humans^28^. However, most previous studies on the association between periodontitis and thyroid dysfunction consist of animal studies, case reports, and retrospective chart review analyses with a limited number of patients^29^. Therefore, this study aimed to evaluate the link between periodontitis and thyroid function using data from Korea National Health and Nutrition Examination Survey (KNHANES), which is a large population-level survey performed by Korean government that provides latest nationally representative data containing credible health data carried out by well-trained staffs^30^.

## Results

**Figure 1:**
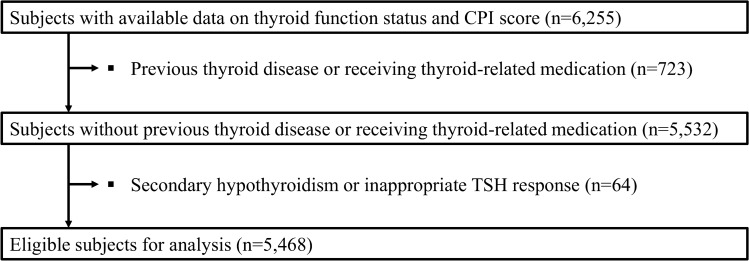
Flow chart of participants included in the study. Abbreviations: CPI: Community Periodontal Index; KHANES, Korea National Health and Nutrition Examination Survey; TSH: thyroid-stimulating hormone.

From a total of 6255 participants, representative of 13,033,409 Koreans, a final sample of 5468 subjects with data on thyroid function status and Community Periodontal Index (CPI) scores were eligible for analyses (Fig. 1). Participants were categorized based on thyroid stimulating hormone (TSH) tertiles. The ranges of serum TSH levels were < 1.76 mIU/L for the first tertile, 1.76–2.83 mIU/L for the second tertile, and > 2.83 mIU/L for the third tertile.

### Baseline characteristics of the study subjects

Table 1 summarizes the baseline characteristics of the study participants according to TSH tertile. Participants presented with a median age of 41 years in all three TSH groups, and 47.6% of the participants were female. A higher proportion of female participants was observed in the highest tertile group than in the other groups (*p* < 0.001). Accordingly, fewer participants smoked and consumed alcohol in the third tertile than in the first or second tertiles (*p* < 0.001 for smoking and *p* = 0.001 for alcohol). No significant differences were observed with regard to fasting glucose, SBP, or total cholesterol among the three groups, though the estimated glomerular filtration rate (eGFR) was slightly higher in the first tertile group than in the other groups (*p* = 0.003). Logarithmically transformed urine iodine concentration and TPOAb positivity were both highest in the third tertile group compared to the other groups (*p* < 0.001 for both urine iodine and TPOAb).

**Table 1:** Baseline characteristics of study participants according to TSH tertiles.

	TSH			*p* value
	1st tertile (n = 1796)	2nd tertile (n = 1811)	3rd tertile (n = 1861)	
Serum TSH level (range)	< 1.76	1.76–2.83	≥ 2.83	
Age (years), median (IQR)	41.0 (28.0–55.0)	41.0 (26.0–56.0)	41.0 (25.0–56.0)	0.375
Sex (female), n (%)	773 (37.1%)	828 (38.2%)	1002 (46.6%)	< 0.001
Body mass index (kg/m^2^), median (IQR)	23.3 (21.0–25.8)	23.4 (21.0–25.6)	23.2 (21.1–25.6)	0.877
Smoking (yes), n (%)	833 (50.4%)	691 (42.1%)	593 (36.4%)	< 0.001
Alcohol (yes), n (%)	1556 (88.1%)	1516 (86.0%)	1532 (85.3%)	0.001
Exercise (yes), n (%)	1571 (88.3%)	1608 (90.0%)	1662 (89.8%)	0.201
Fasting glucose (mg/dL), median (IQR)	93.0 (87.0–101.0)	93.0 (88.0–101.0)	93.0 (88.0–100.0)	0.290
Systolic blood pressure (mmHg), median (IQR)	113.0 (104.0–124.0)	114.0 (105.0–125.0)	113.0 (105.0–124.0)	0.230
Total cholesterol (mg/dL), median (IQR)	182.0 (159.0–206.0)	181.0 (158.0–205.0)	183.0 (160.0–208.0)	0.14
eGFR (mL/min), median (IQR)	93.0 (83.0–105.1)	91.1 (80.2–103.9)	91.3 (80.5–105.7)	0.003
AST (U/L), median (IQR)	19.0 (16.0–24.0)	20.0 (16.0–23.0)	19.0 (16.0–24.0)	0.808
ALT (U/L), median (IQR)	17.0 (12.0–24.0)	17.0 (12.0–24.0)	16.0 (12.0–24.0)	0.199
Log-transformed urine iodine, median (IQR)	2.4 (2.1–2.7)	2.5 (2.2–2.8)	2.6 (2.3–2.9)	< 0.001
Anti-TPO antibody (yes), n (%)	59 (3.1%)	60 (2.7%)	149 (7.9%)	< 0.001
Periodontitis (yes), n (%)	513 (26.5%)	471 (23.1%)	439 (20.9%)	0.003

*AST* aspartate transaminase, *ALT* alanine transaminase, *eGFR* estimated glomerular filtration rate, *IQR* interqurtile range, *TPO* thyroid peroxidase, *TSH* thyroid-stimulating hormone.

### Prevalence of periodontitis according to TSH tertiles

**Figure 2:**
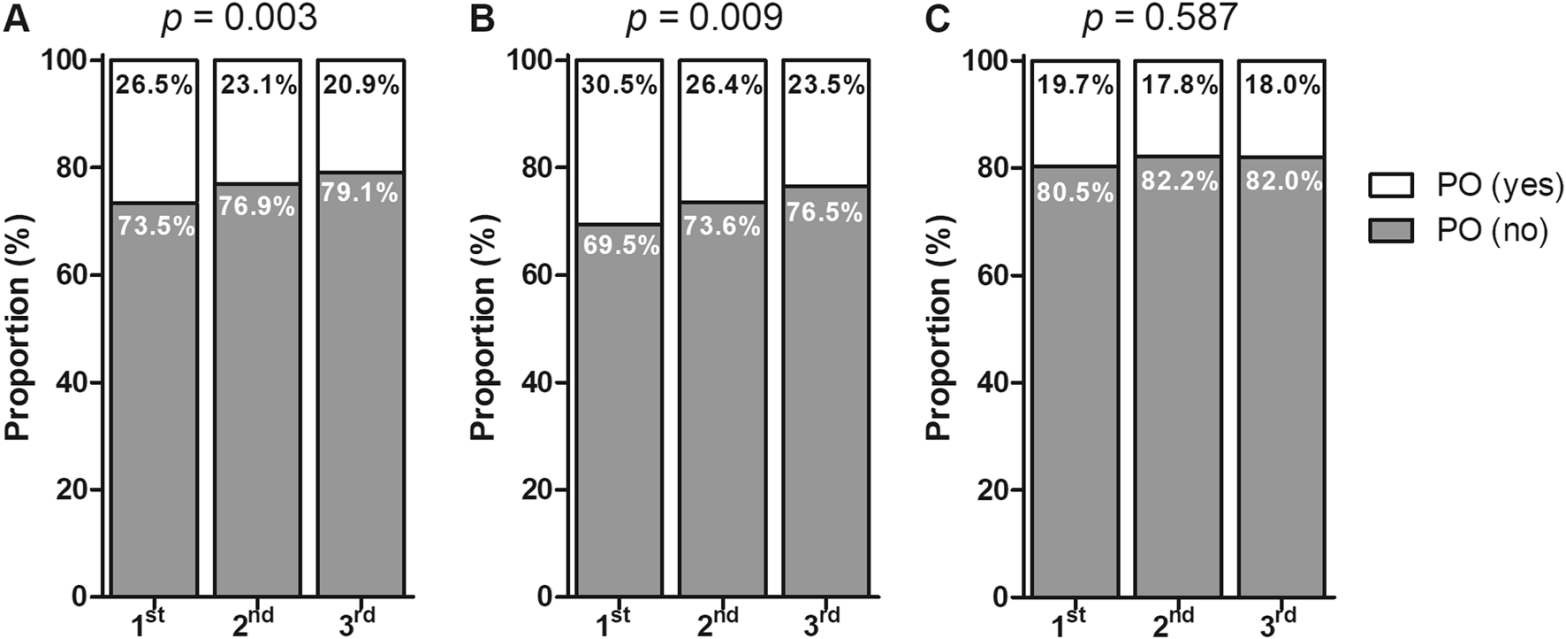
Weighted prevalence of periodontitis according to TSH tertiles in (**A**) all participants, (**B**) males, and (**C**) females. Abbreviations: PO, periodontitis; TSH: thyroid stimulating hormone.

We observed significant differences in the weighted prevalence of periodontitis according to TSH tertile (*p* = 0.003, Fig. 2A); 26.5% of participants in the first TSH tertile presented with periodontitis, whereas this prevalence decreased to 23.1% in the second tertile and 20.9% in the third tertile. Similar results were observed in the subgroup of male participants (*p* = 0.009, Fig. 2B). However, the association between periodontitis and TSH level was not statistically significant among female participants (*p* = 0.587, Fig. 2C).

### Association between periodontitis and serum TSH levels

The impact of serum TSH levels on periodontitis was evaluated using multiple logistic regression analyses (Table 2). In the minimally adjusted model (adjusted for age and sex; model 1), the odds ratio (OR) for periodontitis was 1.34 (95% confidence interval [CI] 1.10–1.63) for the first tertile of TSH compared to the third tertile. The association remained statistically significant after adjusting for age, sex, BMI, smoking, alcohol, exercise, fasting glucose, SBP, total cholesterol, eGFR, AST, and ALT, with an OR of 1.39 (95% CI 1.14–1.71, model 2). Further adjustment for log-transformed urine iodine levels and TPOAb (model 3) showed significantly higher odds for periodontitis among subjects in the lowest TSH tertile group than among those in the third tertile (OR 1.36, 95% CI 1.10–1.68; *p* for trend = 0.005).

**Table 2:** Associations between periodontitis and serum TSH levels.

	1st tertile	2nd tertile	3rd tertile	*p* for trend
Model 1	1.34 (1.11–1.63)	1.09 (0.91–1.31)	1.0 (Ref)	0.003
Model 2	1.39 (1.14–1.71)	1.15 (0.95–1.39)	1.0 (Ref)	0.001
Model 3	1.36 (1.10–1.68)	1.16 (0.96–1.41)	1.0 (Ref)	0.005

Model 1: adjusted for age and sex.

Model 2: adjusted for age, sex, BMI, smoking, alcohol consumption, exercise, fasting glucose, SBP, total cholesterol, eGFR, AST, and ALT.

Model 3: adjusted for age, sex, BMI, smoking, alcohol consumption, exercise, fasting glucose, SBP, total cholesterol, eGFR, AST, ALT, log urine iodine, and TPOAb.

*CI* confidence interval, *OR* odds ratio, *BMI* body mass index, *SBP* systolic blood pressure, *eGFR* estimated glomerular filtration ratio, *AST* aspartate transaminase, *ALT* alanine transaminase, *TPO* thyroid peroxidase, *TSH* thyroid-stimulating hormone.

### Subgroup analyses

**Figure 3:**
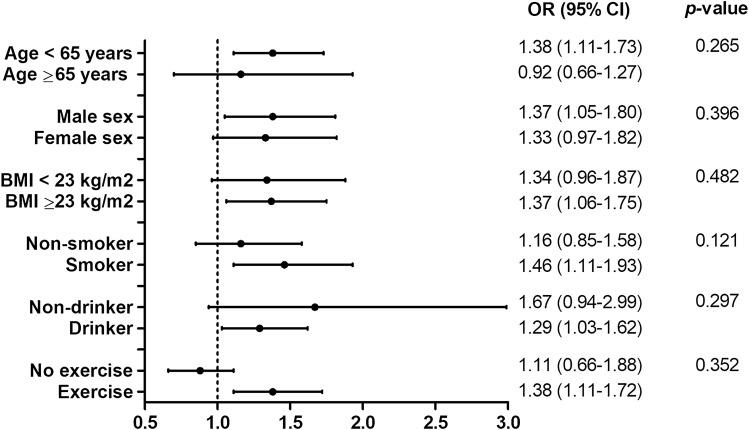
Multivariable adjusted associations between periodontitis and serum TSH levels among participant subgroups. ORs for the 1st tertile group in reference to the 3rd tertile group are presented with 95% CIs. Abbreviations: CI, confidence interval; TSH, thyroid-stimulating hormone.

Figure 3 shows forest plots of the ORs for the first tertile group compared to the third tertile group as well as according to prespecified subgroups defined at baseline. No differences were observed in subgroups stratified by age (< 65 years vs. ≥ 65 years), sex (male vs. female), BMI (< 23 kg/m^2^ vs. ≥ 23 kg/m^2^), smoking (no vs. yes), alcohol intake (no vs. yes), and regular exercise (no vs. yes), with no statistically significant *p*-values for multiplicative interaction.

### Sensitivity analyses

Sensitivity analyses were performed among participants without TPOAb, excessive iodine intake (urine iodine > 300 μg/L according to World Health Organization (WHO) criteria^31^), DM, stroke, coronary artery disease, rheumatoid arthritis, or CKD (defined as eGFR < 60 mL/min). We conducted these sensitivity analyses to examine the robustness of our findings when excluding participants presenting with specific factors affecting thyroid function and/or diseases with well-established associations with periodontitis. These multiple logistic regression analyses using model 33 showed strong associations between TSH levels and periodontitis, with adjusted ORs of 1.33 (1.08–1.65) in participants without TPOAb (Supplementary Table 1), 1.37 (1.04–1.81) in participants with urine iodine ≤ 300 μg/L (Supplementary Table 2), 1.37 (1.10–1.70) in participants without DM (Supplementary Table 3), 1.26 (1.02–1.57) in participants without stroke (Supplementary Table 4), 1.25 (1.01–1.55) in participants without coronary artery disease (Supplementary Table 5), 1.25 (1.01–1.55) in participants without rheumatoid arthritis (Supplementary Table 6), and 1.35 (1.09–1.66) in participants without rheumatoid arthritis (Supplementary Table 7) when comparing participants in the first TSH tertile to those in the third tertile.

## Discussion

This nationwide cohort study of 5468 participants showed that thyroid function was significantly associated with periodontitis. Specifically, lower serum TSH levels were associated with higher prevalence of periodontitis, after adjustment for various confounding factors. This association was consistent within subgroups stratified by age, sex, BMI, smoking status, alcohol consumption, or exercise. Moreover, higher ORs for periodontitis were observed in participants without TPOAb, excessive iodine intake, and diseases well known to be associated with thyroid disorders or periodontitis.

More than half of all-cause mortality worldwide is attributable to inflammation-related diseases such as CVDs, DM, NAFLD, CKD, and autoimmune and neurodegenerative disorders^1,2^. Recent studies have suggested that periodontitis may be a risk factor for these wide range of systemic diseases^8–12,15,16,19^. Periodontitis is a periodontal disorder characterized by both dysbiosis of the oral microbiota and dysregulated inflammatory interactions^32^. Imbalances between proinflammatory and anti-inflammatory cytokines have deleterious effects on tissue destruction in chronic periodontitis^33,34^. These inflammatory processes, along with host immune response, provide the basis for the possible associations between periodontitis and the aforementioned noncommunicable diseases^27,35^. Moreover, studies have observed that inflammatory reactions in periodontitis are related to increased local and systemic oxidative stress and impaired antioxidant capacity, thereby affecting systemic health^23^. Indeed, it is globally recognized that dental care and oral health are key factors for maintaining health and protecting against various systemic diseases^36^. These circumstances highlight the need to evaluate disorders that affect periodontal health. However, the association between thyroid function and periodontitis has not been adequately established, and the present study is yet the largest study examining this association using a nationally representative dataset.

Previous studies regarding the possible link between thyroid function and periodontitis mostly focused on the impact of hypothyroidism in a limited number of patients^37^. Rahangdale et al. reported that clinical parameters associated with periodontal status differed among hypothyroid patients on thyroxine therapy (n = 52) and those in the control group (n = 50)^37^. However, this study was confined to a small group of thyroxine-treated patients with hypothyroidism and did not adhere to an established definition of periodontitis, such as the CPI. Feitosa et al. showed a positive association between hypothyroidism and bone loss in a rat model of ligature-induced periodontitis^38^. In fact, a recent review assessing the relationship between hypothyroidism and periodontitis concluded that, to date, very few high-quality studies have been conducted to determine this association, although the review suggested a modest association between these diseases based on current epidemiological and laboratory-based reports^29^. Our population-based study used TSH levels as an indicator of thyroid function, thereby covering the whole range of thyroid function (including euthyroid and hyperthyroidism). We observed that low TSH levels were rather associated with a higher prevalence of periodontitis, as defined by the CPI criteria.

There is a complex immunopathology underlying the etiology of periodontitis, which is now recognized as a chronic inflammatory disease^39^. Previous studies have investigated the pathogenesis of periodontitis and its association with the inflammatory cytokines IL-1β, IL-4, IL-6, IL-10, interferon-γ (IFN-γ), and TNF-α^39^. Interestingly, various cytokines, including IL-1β, IL-6, IL-10, IFN-γ, and TNF-α, are found in the thyroid follicular cells that augment the inflammatory response^40^. The importance of cytokines and chemokines in the pathogenesis of autoimmune thyroid disorders has been emphasized in recent studies. Recruited T helper 1 (Th1) lymphocytes stimulate IFN-γ and TNF-α production from the thyroid cells, which enhances the secretion of CXCL10, an IFN-γ-inducible Th1 chemokine^41^. This mechanism launches an amplification feedback loop and sustains the autoimmune process^41^. The active phase of hyperthyroidism is associated with increased circulating levels of these chemokines^42^. Moreover, thyroid dysfunction and thyroid autoantibodies have been commonly reported in patients with systemic autoimmune diseases such as rheumatoid arthritis, highlighting the possibility of common pathogenic mechanisms within various autoimmune diseases^43^.

Oxidative stress is a pivotal mechanism underlying the process of inflammation, and a vicious cycle exists between oxidative stress and chronic inflammation^28^. Increased oxidative stress is a characteristic of hyperthyroidism^44^ and hypermetabolic states in hyperthyroidism can cause oxidative tissue injury^45^. Thyroid hormones have been shown to regulate the oxidative system by increasing reactive oxidative species and lowering antioxidant availability^28,46^. It can directly induce DNA damage or enhance nitric oxide synthase gene expression to overproduce nitric oxide^45,47^. Antioxidative status may also be regulated through an increase in the turnover of mitochondrial proteins and mitoptosis^48^. These mechanisms share epidemiological associations with periodontitis—furthermore with systemic inflammatory diseases—and may explain the observed impact of TSH levels on periodontitis in this study. Although bacteria induce periodontitis, previous research indicates that inflammation and oxidative stress may be the main causes of tissue damage progression in periodontitis^49^.

The association between vitamin D levels and autoimmune thyroid disease has been widely studied in recent years^50^. Previous evidence supports the influence of vitamin D deficiency on the increased risk of developing thyroid diseases and/or increased antibody titers^50,51^. Interestingly, a systematic review and meta-analysis demonstrated that circulating vitamin D levels were significantly lower in patients with chronic periodontitis than in healthy controls^52^. These results suggest a possible pathophysiological role of vitamin D in linking thyroid dysfunction and periodontitis. Unfortunately, the present study could not analyze or adjust for vitamin D levels due to the lack of data in KNHANES VI.

The subgroup analyses in the present study clearly demonstrated that associations between TSH levels and periodontitis remained significant regardless of age, sex, BMI, smoking status, alcohol consumption, or exercise (Fig. 3). In addition, results were consistent when sensitivity analyses were performed in subjects without TPOAb or high urine iodine levels (> 300 μg/L). Given the strong and well-established association between periodontitis and rheumatoid arthritis (a classic autoimmune disease in humans^19^), TPOAb, which is the most sensitive marker for detecting autoimmune thyroid disease^53^, could plausibly be related to periodontitis. However, contrary to our expectation, TPOAb had no impact on periodontitis within the current study. This may indicate that thyroid hormone per se has an effect on periodontal inflammation through the above-mentioned mechanisms, although the low TPOAb positivity (4.9%) may have limited the likelihood of reaching statistical significance. In subjects without DM, stroke, coronary artery disease, rheumatoid arthritis, or CKD, the impact of TSH on periodontitis remained significant, supporting the robust association observed between thyroid function and periodontitis.

There are some limitations to address for this study. First, the major limitation lies on the cross-sectional design, and the observed association does not necessarily represent a causal relationship. Longitudinal studies evaluating the temporal association between periodontitis and thyroid function are necessary. Moreover, it would be useful to evaluate if periodontal treatment reduces the risk of thyroid dysfunction. Second, although we adjusted for various possible confounding variables identified through a thorough literature review, there could be residual bias that we were unable to eliminate. Third, the present study included only Korean subjects which prohibit the generalization of the results to the population across different ethnicities. As ethnicity and geographic locations have significant impact on thyroid disease^54^, the results from our study need to be confirmed in other ethnicities. Lastly, periodontitis was defined by CPI criteria only. Despite its longstanding widespread use for screening periodontitis, the CPI requires well-trained dentists, long examination time, and special equipment to perform^55^. Further studies using more recent screening tools with lower bias including half-reduced Centers of Disease Control and Prevention and American Academy of Periodontology case definition or the new 2018 periodontitis case definition by the European Federation of Periodontology and American Association of Periodontology would be valuable^56,57^. Despite these limitations, a substantial strength of this study is the availability of nationally representative data from a large epidemiological study with standardized high-quality clinical and laboratory procedures and a wide range of information about potential confounding factors. Furthermore, the extensive subgroup and sensitivity analyses conducted within this study support the consistency of the present findings.

In conclusion, TSH levels were significantly associated with periodontitis, independent of various risk factors for periodontitis. Given that numerous systemic diseases have been linked to periodontal disease, our findings may provide important guidance for clinicians to evaluate periodontal disease in patients with thyroid dysfunction.

## Methods

### Study design and population

Data were obtained from the KNHANES, a nationwide cross-sectional survey conducted by the Korea Centers for Disease Control and Prevention to provide national health, diet, and nutrition data representing the civilian, non-institutionalized Korean population. This study was conducted with data from version VI of the survey, conducted from 2013 to 2015. The research subjects were selected using two-stage stratified cluster sampling of the population as well as housing census data. Data were collected via household interviews and standardized physical examinations. The institutional review board of the Korea Centers for Disease Control and Prevention approved the protocol of the KNHANES (IRB approval number: 2013-07CON-03-4C, 2013-12EXP-03-5C, and 2015-01-02-6C). The study was performed in accordance with the relevant guidelines and regulations. Written informed consent was obtained from each participant before the survey, and secondary anonymized data were used in the analyses. The anonymized KNHANES database is publicly available at http://knhanes.cdc.go.kr/knhanes/eng.

This study initially selected participants with data on thyroid function status and CPI scores (n = 6255, Fig. 1). Participants with previous thyroid disease, those receiving thyroid-related medications were excluded from the analyses (n = 723). Participants with an inappropriate TSH response (free T4 > 1.76 ng/mL and TSH ≥ 0.62 mIU/L) or secondary hypothyroidism (free T4 < 0.89 ng/mL and TSH ≤ 6.8 mIU/L) were excluded as well (n = 64), as these findings are very unusual and generally require repeated thyroid function testing. A total of 5468 participants were included in the final study. The study protocol was approved by the Institutional Review Board of Korea University.

### Laboratory test

Thyroid function tests were conducted in one-third of all KNHANES VI participants, (approximately 2400 persons aged ≥ 10 years annually). The sum of 3 years of testing represents the entire population of Korea. Serum TSH was measured using an electrochemiluminescence immunoassay (E-TSH kit, Roche Diagnostics, Mannheim, Germany), and the TSH reference interval was determined to be between the 2.5th and 97.5th percentile of serum TSH levels measured in the reference population (0.62–6.86 mIU/L)^58^. TSH levels were logarithmically transformed to normalize the distribution. In this study, we used the TSH levels to represent the thyroid function as TSH is the most sensitive marker of thyroid function, influenced by minute changes in free T4 concentrations^59^. Serum free T4 was also measured through an electrochemiluminescence immunoassay (E-Free T4 kit, Roche Diagnostics, Mannheim, Germany), with a reference range of 0.89–1.76 ng/mL. Anti-thyroid peroxidase antibody (TPOAb) levels were measured using an E-Anti-TPO kit (Cobas e 801; Roche Diagnostics) with a reference range of 0–34 IU/mL. Urine iodine concentrations were measured using an inductively coupled plasma mass spectrometer (ICP-MS; Perkin Elmer ICP-MS, Waltham, MA, USA) with an iodine standard (Inorganic Venture, Christiansburg, VA, USA).

### Definition of periodontitis

An oral health examination was performed within the KNHANES by calibrated dentists. Participants’ periodontal condition was assessed using the CPI, which was developed by the WHO^60^. Periodontitis was defined as a CPI code ≥ 3, indicating that at least one site had a > 3.5-mm pocket in the index tooth (11, 16, 17, 26, 27, 31, 36, 37, 46, and/or 47 according to the *Federation Dentaire Internationale* system).

### Statistical analysis

R version 3.4.0 software, the R libraries survey (RODBC), and Cairo were used for data analysis (R Foundation for Statistical Computing, Vienna, Austria; available at http://www.R-project.org). All statistics were calculated using sample weights assigned to the sample participants within the KNHANES. The sample weights were constructed to attain unbiased estimates representing the entire Korean population, with consideration of the stratified multistage probability sampling design of each survey year. Continuous variables were presented as medians with interquartile ranges (IQRs) and categorical variables were presented as numbers with percentages. The distributions of continuous and categorical variables were compared using the Kruskal–Wallis and chi-square tests, respectively. Associations between periodontitis and TSH levels were analyzed using multivariable logistic regression models adjusted for potential confounding factors, including age, sex, body mass index (BMI), smoking, alcohol intake, exercise, fasting glucose levels, systolic blood pressure (SBP), total cholesterol, eGFR (calculated by the MDRD equation), AST, ALT, log urine iodine, and TPOAb. Sensitivity analyses were performed by assessing associations between periodontitis and serum TSH levels among participants without specific factors affecting thyroid function, as well as among participants without diseases reported to be related to periodontitis within previous studies. Associations were considered statistically significant at a *p* value of < 0.05.

## Supplementary Information

Supplementary Tables.

## Data Availability

The anonymized KNHANES database is publicly available at http://knhanes.cdc.go.kr/knhanes/eng.
