# Supplementary material for: Implication of thyroid function in periodontitis: a nationwide population-based study
Source: Sci Rep. 2021 Nov 11;11:22127. doi: 10.1038/s41598-021-01682-9 (PMC8586139; doi:10.1038/s41598-021-01682-9)
Supplement: Supplementary file 1 — Supplementary Tables. [file 41598_2021_1682_MOESM1_ESM.docx]

**Implication of Thyroid Function in Periodontitis: A Nationwide Population-based Study**

**Eyun Song, Min Jeong Park, Jung A Kim, Eun Roh, Ji Hee Yu, Nam Hoon Kim, Hye Jin Yoo, Ji A. Seo, Sin Gon Kim, Nan Hee Kim, Sei Hyun Baik, and Kyung Mook Choi**

**Supplementary Table 1.** Association between periodontitis and serum TSH levels in participants without TPOAb.

|  | 1^st^ tertile | 2^nd^ tertile | 3^rd^ tertile | *p* for trend |
| --- | --- | --- | --- | --- |
| Model 1 | 1.35 (1.10–1.64) | 1.09 (0.90–1.32) | 1.0 (Ref) | 0.003 |
| Model 2 | 1.37 (1.11–1.69) | 1.14 (0.93–1.39) | 1.0 (Ref) | 0.003 |
| Model 3 | 1.33 (1.08–1.65) | 1.14 (0.94–1.40) | 1.0 (Ref) | 0.009 |

Model 1: adjusted for age and sex.

Model 2: adjusted for age, sex, BMI, smoking, alcohol consumption, exercise, fasting glucose, SBP, total cholesterol, eGFR, AST, and ALT.

Model 3: adjusted for age, sex, BMI, smoking, alcohol consumption, exercise, fasting glucose, SBP, total cholesterol, eGFR, AST, ALT, and log urine iodine.

Abbreviations: CI, confidence interval; OR, odds ratio; BMI, body mass index; SBP, systolic blood pressure; eGFR, estimated glomerular filtration ratio; AST, aspartate transaminase; ALT, alanine transaminase; TPO, thyroid peroxidase; TPOAb: anti-thyroid peroxidase antibody; TSH: thyroid-stimulating hormone.

**Supplementary Table 2.** Association between periodontitis and serum TSH levels in participants with urine iodine ≤300 μg/L.

|  | 1^st^ tertile | 2^nd^ tertile | 3^rd^ tertile | *p* for trend |
| --- | --- | --- | --- | --- |
| Model 1 | 1.40 (1.08–1.81) | 1.16 (0.89-0.57) | 1.0 (Ref) | 0.012 |
| Model 2 | 1.42 (1.08–1.86) | 1.16 (0.88–1.53) | 1.0 (Ref) | 0.010 |
| Model 3 | 1.37 (1.04–1.81) | 1.18 (0.89–1.56) | 1.0 (Ref) | 0.024 |

Model 1: adjusted for age and sex.

Model 2: adjusted for age, sex, BMI, smoking, alcohol consumption, exercise, fasting glucose, SBP, total cholesterol, eGFR, AST, and ALT.

Model 3: adjusted for age, sex, BMI, smoking, alcohol consumption, exercise, fasting glucose, SBP, total cholesterol, eGFR, AST, ALT, and TPOAb.

Abbreviations: CI, confidence interval; OR, odds ratio; BMI, body mass index; SBP, systolic blood pressure; eGFR, estimated glomerular filtration ratio; AST, aspartate transaminase; ALT, alanine transaminase; TPO, thyroid peroxidase; TPOAb: anti-thyroid peroxidase antibody; TSH: thyroid-stimulating hormone.

**Supplementary Table 3.** Association between periodontitis and serum TSH Levels in participants without diabetes mellitus.

|  | 1^st^ tertile | 2^nd^ tertile | 3^rd^ tertile | *p* for trend |
| --- | --- | --- | --- | --- |
| Model 1 | 1.34 (1.09–1.64) | 1.13 (0.93–1.37) | 1.0 (Ref) | 0.006 |
| Model 2 | 1.39 (1.12–1.71) | 1.19 (0.97–1.46) | 1.0 (Ref) | 0.002 |
| Model 3 | 1.37 (1.10–1.70) | 1.20 (0.98–1.47) | 1.0 (Ref) | 0.005 |

Model 1: adjusted for age and sex.

Model 2: adjusted for age, sex, BMI, smoking, alcohol consumption, exercise, fasting glucose, SBP, total cholesterol, eGFR, AST, and ALT.

Model 3: adjusted for age, sex, BMI, smoking, alcohol consumption, exercise, fasting glucose, SBP, total cholesterol, eGFR, AST, ALT, log urine iodine, and TPOAb.

Abbreviations: CI, confidence interval; OR, odds ratio; BMI, body mass index; SBP, systolic blood pressure; eGFR, estimated glomerular filtration ratio; AST, aspartate transaminase; ALT, alanine transaminase; TPO, thyroid peroxidase; TPOAb: anti-thyroid peroxidase antibody; TSH: thyroid-stimulating hormone.

**Supplementary Table 4.** Association between periodontitis and serum TSH levels in subjects without history of stroke.

|  | 1^st^ tertile | 2^nd^ tertile | 3^rd^ tertile | *p* for trend |
| --- | --- | --- | --- | --- |
| Model 1 | 1.29 (1.05 – 1.58) | 1.10 (0.91 – 1.34) | 1.0 (Ref) | 0.015 |
| Model 2 | 1.30 (1.06 – 1.61) | 1.16 (0.95 – 1.41) | 1.0 (Ref) | 0.014 |
| Model 3 | 1.26 (1.02 – 1.57) | 1.16 (0.95 – 1.41) | 1.0 (Ref) | 0.037 |

Abbreviation: CI, confidence interval; OR, odds ratio.

Model 1: adjusted for age and sex

Model 2: adjusted for age, sex, BMI, smoking, alcohol, exercise, fasting glucose, SBP, total cholesterol, eGFR, AST, ALT

Model 3: adjusted for age, sex, BMI, smoking, alcohol, exercise, fasting glucose, SBP, total cholesterol, eGFR, AST, ALT, log urine iodine, TPOAb

**Supplementary Table 5.** Association between periodontitis and serum TSH levels in subjects without history of coronary artery disease.

|  | 1^st^ tertile | 2^nd^ tertile | 3^rd^ tertile | *p* for trend |
| --- | --- | --- | --- | --- |
| Model 1 | 1.27 (1.04 – 1.56) | 1.12 (0.92 – 1.35) | 1.0 (Ref) | 0.019 |
| Model 2 | 1.29 (1.04 – 1.59) | 1.17 (0.96 – 1.43) | 1.0 (Ref) | 0.020 |
| Model 3 | 1.25 (1.01 – 1.55) | 1.18 (0.97 – 1.44) | 1.0 (Ref) | 0.044 |

Abbreviation: CI, confidence interval; OR, odds ratio.

Model 1: adjusted for age and sex

Model 2: adjusted for age, sex, BMI, smoking, alcohol, exercise, fasting glucose, SBP, total cholesterol, eGFR, AST, ALT

Model 3: adjusted for age, sex, BMI, smoking, alcohol, exercise, fasting glucose, SBP, total cholesterol, eGFR, AST, ALT, log urine iodine, TPOAb

**Supplementary Table 6.** Association between periodontitis and serum TSH levels in participants without rheumatoid arthritis.

|  | 1^st^ tertile | 2^nd^ tertile | 3^rd^ tertile | *p* for trend |
| --- | --- | --- | --- | --- |
| Model 1 | 1.28 (1.04 – 1.56) | 1.14 (0.94 – 1.38) | 1.0 (Ref) | 0.018 |
| Model 2 | 1.28 (1.04 – 1.58) | 1.19 (0.98 – 1.45) | 1.0 (Ref) | 0.020 |
| Model 3 | 1.25 (1.01 – 1.55) | 1.20 (0.99 – 1.46) | 1.0 (Ref) | 0.045 |

Model 1: adjusted for age and sex.

Model 2: adjusted for age, sex, BMI, smoking, alcohol consumption, exercise, fasting glucose, SBP, total cholesterol, eGFR, AST, and ALT.

Model 3: adjusted for age, sex, BMI, smoking, alcohol consumption, exercise, fasting glucose, SBP, total cholesterol, eGFR, AST, ALT, log urine iodine, and TPOAb.

Abbreviations: CI, confidence interval; OR, odds ratio; BMI, body mass index; SBP, systolic blood pressure; eGFR, estimated glomerular filtration ratio; AST, aspartate transaminase; ALT, alanine transaminase; TPO, thyroid peroxidase; TPOAb: anti-thyroid peroxidase antibody; TSH: thyroid-stimulating hormone.

**Supplementary Table 7.** Association between periodontitis and serum TSH levels in participants without chronic kidney disease (eGFR <60 mL/min).

|  | 1^st^ tertile | 2^nd^ tertile | 3^rd^ tertile | *p* for trend |
| --- | --- | --- | --- | --- |
| Model 1 | 1.33 (1.10–1.62) | 1.09 (0.91–1.32) | 1.0 (Ref) | 0.004 |
| Model 2 | 1.37 (1.12–1.68) | 1.15 (0.95–1.40) | 1.0 (Ref) | 0.002 |
| Model 3 | 1.35 (1.09–1.66) | 1.17 (0.96–1.42) | 1.0 (Ref) | 0.006 |

Model 1: adjusted for age and sex.

Model 2: adjusted for age, sex, BMI, smoking, alcohol consumption, exercise, fasting glucose, SBP, total cholesterol, eGFR, AST, and ALT.

Model 3: adjusted for age, sex, BMI, smoking, alcohol consumption, exercise, fasting glucose, SBP, total cholesterol, eGFR, AST, ALT, log urine iodine, and TPOAb.

Abbreviations: CI, confidence interval; OR, odds ratio; BMI, body mass index; SBP, systolic blood pressure; eGFR, estimated glomerular filtration ratio; AST, aspartate transaminase; ALT, alanine transaminase; TPO, thyroid peroxidase; TPOAb: anti-thyroid peroxidase antibody; TSH: thyroid-stimulating hormone.
